# Using Regular High-Quality Serosurveys to Identify and Close National Immunity Gaps—Measles and Rubella Elimination in Japan

**DOI:** 10.3390/vaccines12080939

**Published:** 2024-08-22

**Authors:** Tomimasa Sunagawa, Yusuke Kobayashi, Yoshihiro Takashima, Hajime Kamiya, Tomoe Shimada, Kazutoshi Nakashima, Satoru Arai, Kiyosu Taniguchi, Keiko Tanaka-Taya, Nobuhiko Okabe

**Affiliations:** 1 National Institute of Infectious Diseases, Tokyo 162-8640, Japan; 2 Osaka University Center for Infectious Disease Education and Research, Osaka 565-0871, Japan; 3 Graduate School of Medicine, Mie University, Tsu 514-8507, Japan; 4 Department of Health Science, Faculty of Sports and Health Science, Daito Bunka University, Tokyo 175-8571, Japan; 5 Department of Pediatrics, Mie National Hospital, National Hospital Organization, Tsu 514-0125, Japan; 6 Kanagawa Prefectural Institute of Public Health, Kanagawa 253-0087, Japan; 7 Kawasaki City Institute for Public Health, Kanagawa 210-0821, Japan

**Keywords:** measles, rubella, elimination, immunity gaps, serological surveillance, SIAs

## Abstract

In Japan, periodic measles outbreaks occurred mainly among young children under the routine immunization program with one dose of the measles-containing vaccine (MCV). A second dose of MCV was introduced in 2006. During a nationwide measles resurgence in 2007–2008, the most affected age group was teenagers. The national serological surveillance for vaccine-preventable diseases made it clear that there was a measles immunity gap among teenagers who had not received a second dose of MCV. To fill this immunity gap, nationwide non-selective supplementary immunization activities (SIAs) were carried out as a five-year program from April 2008 to March 2013 by providing an opportunity to be vaccinated with the measles and rubella vaccine during the first year of junior high school (12–13 years old) and the last year of high school (17–18 years old). The SIA was conducted with the strong involvement of local governments in charge of vaccination delivery and collaboration between the health and education sectors. Japan was verified as achieving measles elimination in 2015 and this has been sustained to date. The challenge of rubella elimination following a similar strategy of a serological diagnosis of an immunity gap and targeted vaccination is also discussed.

## 1. Introduction

### 1.1. Objectives of This Review

Measles and rubella are viral and representative vaccine-preventable diseases [1]. Measles can lead to serious complications such as pneumonia, encephalitis, and death is airborne and spread by respiratory droplets. Rubella is generally a milder illness than measles but it poses a significant risk to pregnant women and can cause congenital rubella syndrome (CRS), leading to serious birth defects such as heart abnormalities, cataracts, and deafness. There are highly effective vaccines for both measles and rubella, and they are generally used as a combination vaccine [2,3].

The world has had the shared goal of eliminating the endemic transmission of measles and rubella viruses, which will also result in the elimination of CRS. For example, the Region of the Americas (PAHO), consisting of 35 member states, declared measles elimination in 2016 (two of these countries lost their elimination certification in 2018, but only one is currently pending reverification), and rubella elimination in 2015, with remaining to date. This has been achieved through the implementation of PAHO-recommended surveillance and immunization strategies [4]. In the Western Pacific Region consisting of 27 member states including Japan, as of May 2024, 8 countries and regions had been verified as having achieved and sustained measles elimination, with 7 verified for rubella elimination [5].

We describe and discuss how Japan, as a country using national serological surveillance to routinely identify measles immunity gaps, planned strategies to fill them and then monitored the impact of these strategies. We consider the impact on achieving and sustaining measles elimination. We also analyze and discuss the application of the same approach to achieving rubella elimination.

### 1.2. Immunization Program in Japan

In Japan, while the Ministry of Health, Labor, and Welfare has overall responsibility for the national immunization program [1], implementation of the program is principally carried out by municipalities (local governments) (*n* = 1718) based on the Immunization Act [6]. Currently, the majority of vaccines are administered at local medical facilities where physicians contracted by municipalities provide vaccination services. The physician records the date, dose, and lot number of vaccines administered in the Maternal and Child Health Handbook and sends the filled vaccine screening questionnaire to the local government so that the medical facility can be reimbursed. The local government retains the form and calculates the local vaccination coverage.

### 1.3. Serological Surveillance Programme in Japan

Serological surveillance in Japan was launched in 1962 and it has been called the National Epidemiological Surveillance of Vaccine-Preventable Diseases (NESVPD) since the enforcement of the Infectious Diseases Control Law in 1999. Local public health institutes participate each fiscal year (hereafter FY; from April to March in Japan), collaborating with health facilities to collect blood samples according to the numbers of targeted areas and subjects, with serological analysis for VPDs such as measles and rubella. The results are to be shared with NIID and feedback is provided to the public [7]. For example, in FY 2023, the survey was conducted in 23 prefectures and 23 areas with 4554 people targeted for measles serology, with a total of 18 prefectures, 24 areas, and 5982 people targeted for rubella serology [7].

### 1.4. Measles and Rubella Control with Vaccination in Japan, 1979–2005

Under the routine immunization program, from October 1978 to March 1995, the measles vaccine (MV) was given to both boys and girls aged 12–72 months. From August 1977 to March 1995, the rubella vaccine (RV) was given to only girls aged 12–15 years. From April 1995 to March 2006, the measles and rubella vaccine (MRV) was given to children (both boys and girls) aged 12 to 90 months [8,9]. Accurate coverage was estimated from 1995 and, since that time, MRV coverage has been maintained above 90%. While the targeted age range for MRV vaccination in Japan was wide, local clinicians were encouraged to administer the measles-containing vaccine (MCV) as soon as possible after the child’s first birthday, and MCV coverage at the first year of age has increased since the early 2000s.

**Figure 1:**
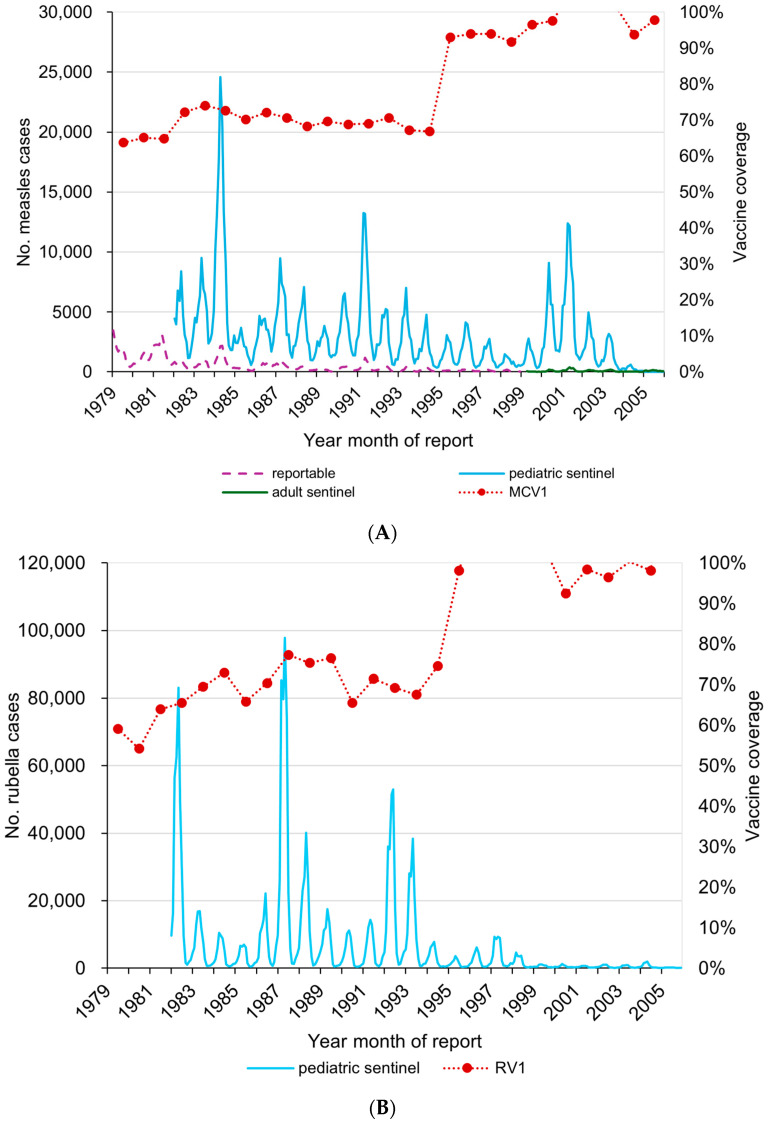
Measles and rubella vaccination * and aggregated numbers from sentinel sites ^†^ in Japan, 1979–2005. (**A**) Measles. (**B**) Rubella. MV, measles vaccine; RV, rubella vaccine. * Vaccine coverage = number of people immunized (the Report on Regional Public Health Services)/total population of standard immunization age period (as of October 1). The numerator is the number of people who received vaccinations out of the total number of people eligible for vaccinations, while the denominator is the population newly eligible for vaccinations each year, so the coverage rate may exceed 100%. ^†^ Since 1947, measles has been a reportable disease for surveillance. Since 1983, measles has been reported as pediatric sentinel surveillance. Under the current law (Infectious Diseases Control Law), measles and rubella have been reported as sentinel surveillance from 1999 to 2007. During 1999–2008, measles in adults was reported from hospitalized sentinel sites.

In 1947, measles became a reportable disease for surveillance in Japan. From 1983 to 2007, the occurrence of both measles and rubella was monitored by the pediatric sentinel surveillance system involving around 3000 pediatric hospitals and clinics [8,9]. From 1999 to 2007, sentinel surveillance for adult measles was also operational [8]. There were several measles resurgences recorded, with cases from sentinel sites exceeding 50,000 cases in a number of years (e.g., 1983, 1984, 1987, and 1991) (Figure 1A) [10,11].

### 1.5. Measles Outbreak in 2007–2008

In Japan, the second dose of MRV (MRV-2) was introduced into the routine vaccination program in 2006 for children aged 5–7 years with the first dose of MRV (MRV-1) given to children aged 12–24 months. While MRV-1 had been given with high coverage since 1995 (>90%) and MRV-2 was introduced with high coverage (80%) in 2006, the country experienced a nationwide measles resurgence in 2007–2008 [1,8], which triggered the development and implementation of a national strategy and plan of action for measles elimination in Japan (“Guidelines for the Prevention of Specific Infectious Diseases Related to Measles”, hereinafter “Guidelines”) [12].

### 1.6. Measles Elimination Program

The Guidelines were prepared to address and overcome several critical challenges in measles elimination in Japan, which included (1) the existence of large immunity gaps among adolescents; (2) the limited production capacity of domestic MCV, where around 1.2 million doses were produced by three domestic vaccine manufactures annually in 2005 for the routine MCV-1 and 2.3 million doses of MCV-1 and MCV-2 in 2006 [13]; (3) insufficient information on how to implement elimination-focused vaccination programs among municipalities; (4) limited experiences and technical capacity of local government to conduct nationwide case-based measles and rubella surveillance including active case investigation started in 2008; and (5) insufficient awareness of the importance of preventing measles in schools and immunization for high-school-aged students.

To overcome those challenges, the Guidelines includes strategies and activities to (1) strengthen routine vaccination with MRV-1 (for children aged 12–24 months) and MRV-2 (for children aged 5–6 years), with cohorts born after 2000 specifically targeted; (2) identify birth cohorts with higher susceptibility to measles virus infection and transmission using the national serological surveillance system; (3) develop and conduct a nationwide non-selective catch-up measles and rubella supplemental immunization activity (MR-SIA) to fill large immunity gaps with full consideration of the national capacity for vaccine supply; (4) achieve and maintaine high vaccination coverage in all municipalities; (5) enhance case-based surveillance with aggressive case and outbreak investigations to detect and report all measles cases with laboratory confirmation and virus genotyping; (6) clearly determine the roles and responsibilities of national and local governments in planning and implementing elimination activities; (7) fully involve the education sector for the vaccination of adolescents; (8) strengthen the outbreak response by local government in collaboration with the NIID; and (9) sensitize the public.

### 1.7. Progress and Achievements

**Figure 2:**
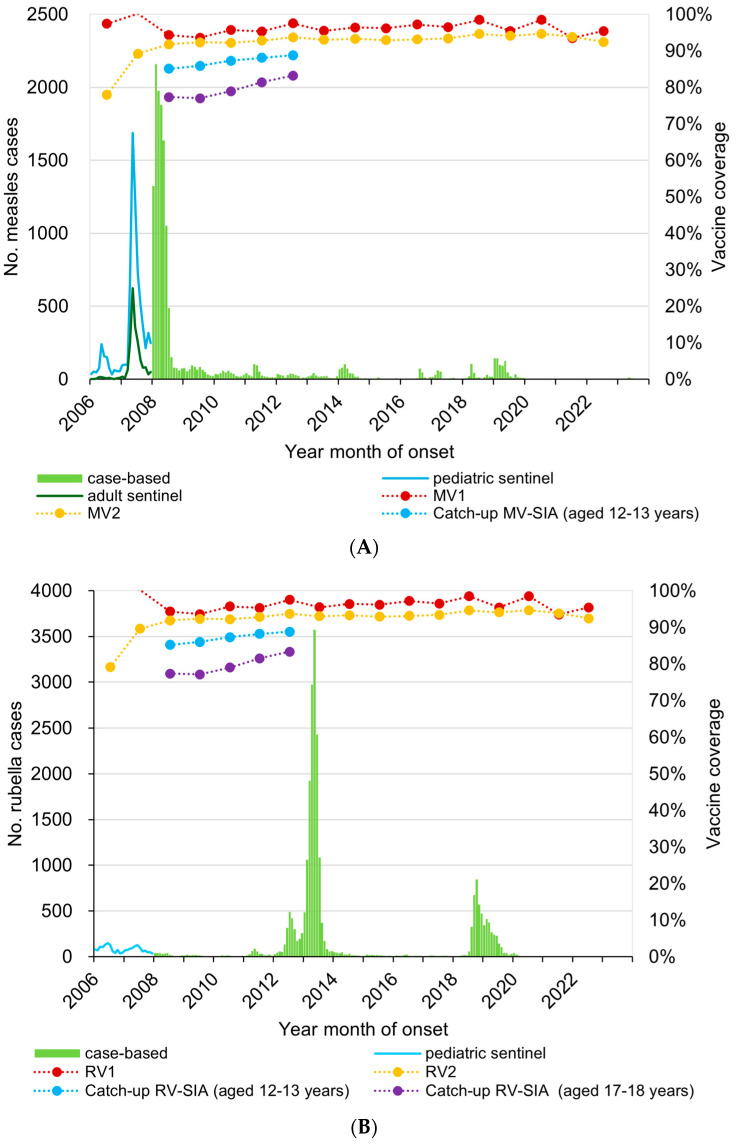
Measles and rubella vaccination * and occurrence ^†^ in Japan, 2006–2022. (**A**) Measles. (**B**) Rubella. MV, measles vaccine; RV, rubella vaccine. * Vaccine coverage = number of people immunized (the Report on Regional Public Health Services)/total population of standard immunization age period (as of October 1). The numerator is the number of people who received vaccinations out of the total number of people eligible for vaccinations, while the denominator is the population newly eligible for vaccinations each year, so the coverage rate may exceed 100%. ^†^ Since 2008, all measles cases have been reported.

From 2008 to 2012, strategies in the Guidelines were intensively and rigorously implemented across the country, resulting in the last detection of endemic measles virus (genotype D5) in 2010 [14] and the confirmation of the interruption of endemic virus transmission in 2012. In 2015, the WHO’s Western Pacific Regional Verification Commission for Measles and Rubella Elimination (RVC) verified Japan to have achieved measles elimination [15]. Since then, measles elimination has been successfully sustained even under the pressure of multiple measles virus importation from endemic countries, particularly during the regional measles resurgence in the Western Pacific in 2013–2016 and the global measles resurgence in 2018–2019 (Figure 2) [10,11,16,17].

## 2. Materials and Methods

This study is a narrative review. We have used measles and rubella surveillance data, which are publicly available, from the National Epidemiological Surveillance of Infectious Diseases (NESID), serological data from the National Epidemiological Surveillance of Vaccine-Preventable Diseases (NESVPD), and technical guidelines prepared by the National Institute of Infectious Diseases (NIID) and issued by the Ministry of Health, Labor, and Welfare (MHLW).

NESID mainly consists of (1) pathogen reporting (laboratory-based surveillance) and (2) patient reporting [18]. For NESVPD [7], local public health institutes collaborate and share results with NIID.

We analyzed and characterized a measles resurgence in 2008, the transmission of measles virus in 2013–2022, and rubella resurgences in 2013–2014 and 2018–2019 and reviewed the impact of strategies and activities carried out from 2008 described in the National Strategy and Plan of Action for Measles Elimination on measles elimination and rubella elimination in Japan.

As this study utilized publicly available surveillance data with only aggregated information, ethical approval was not required.

## 3. Results

### 3.1. Measles Outbreak in 2007–2008

**Figure 3:**
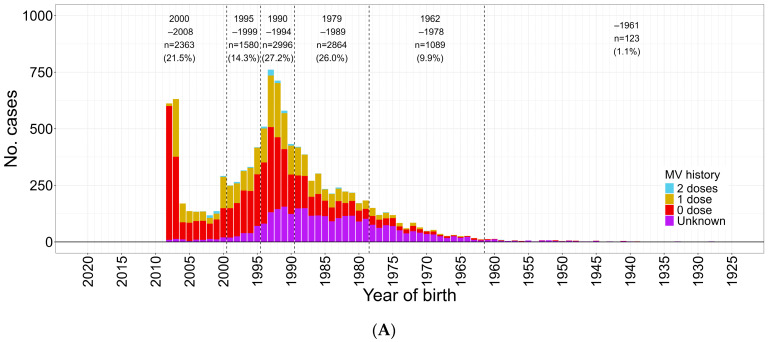

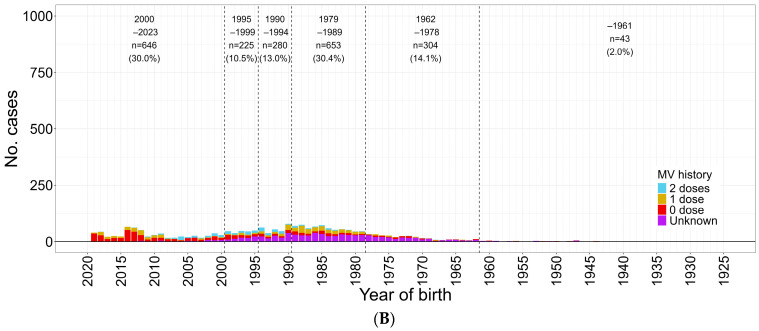
Measles cases (confirmed) by year of birth and vaccination history during measles outbreak (January–December 2008 and January 2013–December 2023); (**A**) January–December 2008 (*n* = 11,015). (**B**) January 2013–December 2023 (*n* = 2151). MV, measles vaccine.

Before 2007, measles surveillance was carried out at sentinel sites for children and adults (rubella only for children), and a major measles outbreak starting in 2007 was obvious from both sentinel surveillance systems. With the transition to nationwide case-based surveillance for both measles and rubella in 2008, we focus our description on 2008. There were 11,013 measles cases reported from January to December 2008. Among them, 2363 (21.5%), 1580 (14.3%), and 2866 (27.2%) were reported from the cohorts born in FY 2000–2008, FY 1995–1999, and FY 1990–1994, respectively, with 63.0% of reported cases aged younger than 15 years (Figure 2A and Figure 3A) [10,19].

### 3.2. Determination of Immunity Gaps to Be Filled

**Figure 4:**
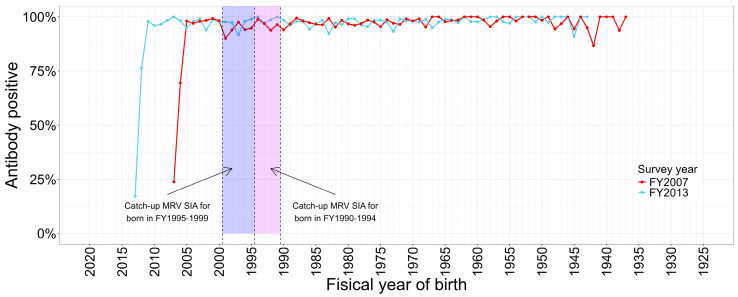
Measles antibody positive rate (PA ≥ 1:16) by year of birth in 2007 and 2013. MRV, measles and rubella vaccine; SIA, supplementary immunization activity; FY, fiscal year.

NESVPD revealed and confirmed that there was a significant measles immunity gap among the cohorts born in FY 1995–1999 and FY 1990–1994 compared with other birth cohorts (Figure 4) [7].

### 3.3. Immunization Strategy 2008–2012 and 2013 to Date

To fill the immunity gap among the cohorts born in FY 1990–1999 and establish high population immunity among all birth cohorts born after FY 2000, the immunization strategy developed was to (1) double the production of domestic MRV from 2.3 million in 2006 to 4.7 million per year from 2008 to 2012 to cover 20 birth cohorts with either routine childhood vaccination or the catch-up MR-SIA over five years; and (2) provide MRV to four birth cohorts from 2008 to 2012 through (i) routine MRV-1 at the age of 12–24 months (the cohorts born in 2008–2012), (ii) routine MRV-2 at the age of 5–6 years (the cohorts born in 2000–2007), (iii) the catch-up MR-SIA at the age of 12–13 years (the first year at junior high school) (the cohorts born between 1995 and 1999), and (iv) the catch-up MR-SIA at the age of 17–18 years (the last year at the senior high school) (the cohorts born between 1990 and 1994).

Routine MRV-1 and MRV-2 coverage was achieved and sustained at >90% (Figure 2A,B). The proportion of prefectures (*n* = 47) with catch-up coverage at the age of 12–13 years and 17–18 years >85% continued to increase from 76.6% to 87.2% and 23.4% to 68.1%, respectively, from 2008 to 2012. More than 90% of prefectures have maintained the coverage of routine MRV-1 and MRV-2 >90% from 2010 to date (Table 1) [20].

Vaccination coverage of the 12–13-year-old and 17–18-year-old SIAs increased every year, starting at 85.1% and 77.3% in 2008, respectively, and ending at 88.8% and 83.2% in 2012, respectively, through the enhanced engagement of local governments and local medical associations and the support of schools, teachers, and parents (Table 2) [13,20].

### 3.4. Impact of the Immunization Strategy on Measles

**Figure 5:**
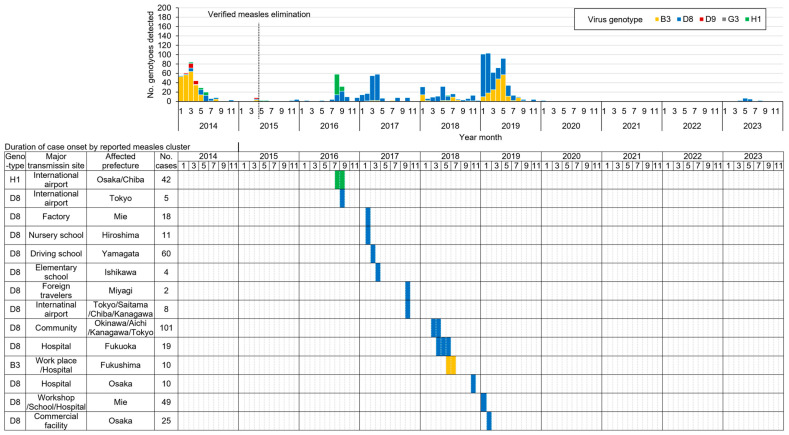
Detected measles virus genotypes and reported measles clusters, 2014–2023.

The last chain of endemic measles transmission due to the genotype D5 virus was confirmed to have been interrupted in May 2010. Since then and up to the end of 2023, Japan has experienced multiple importations of genotypes D9, H1, D8, and B3 measles viruses, particularly between 2016 and 2019. Some of these resulted in small clusters of import-related measles cases among inadequately vaccinated young adults, but rigorous case and outbreak investigation confirmed that each was controlled within a relatively short period with four or fewer generations of virus transmission in limited geographical areas (e.g., within a prefecture) without large-scale outbreaks developing (Figure 5) [17,21].

The Catch-up MR SIA at the age of 12–13 years and 17–18 years conducted in 2008–2012 with enhanced routine MRV-1 and MRV-2 coverage from 2006 established high population immunity among the cohorts born after 1990 and enabled Japan to achieve and sustain measles elimination and prevent the re-establishment of endemic transmission due to imported measles virus.

### 3.5. Impact of the Immunization Strategy on Rubella

**Figure 6:**
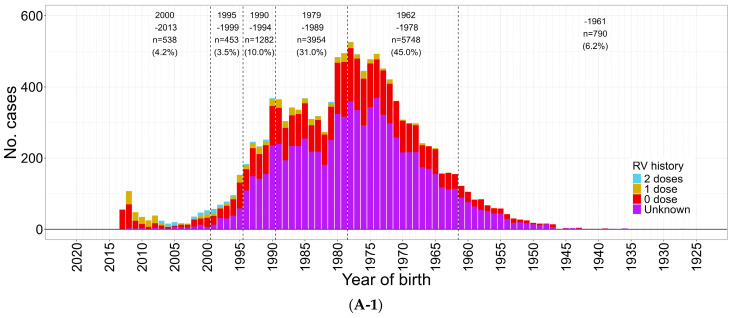

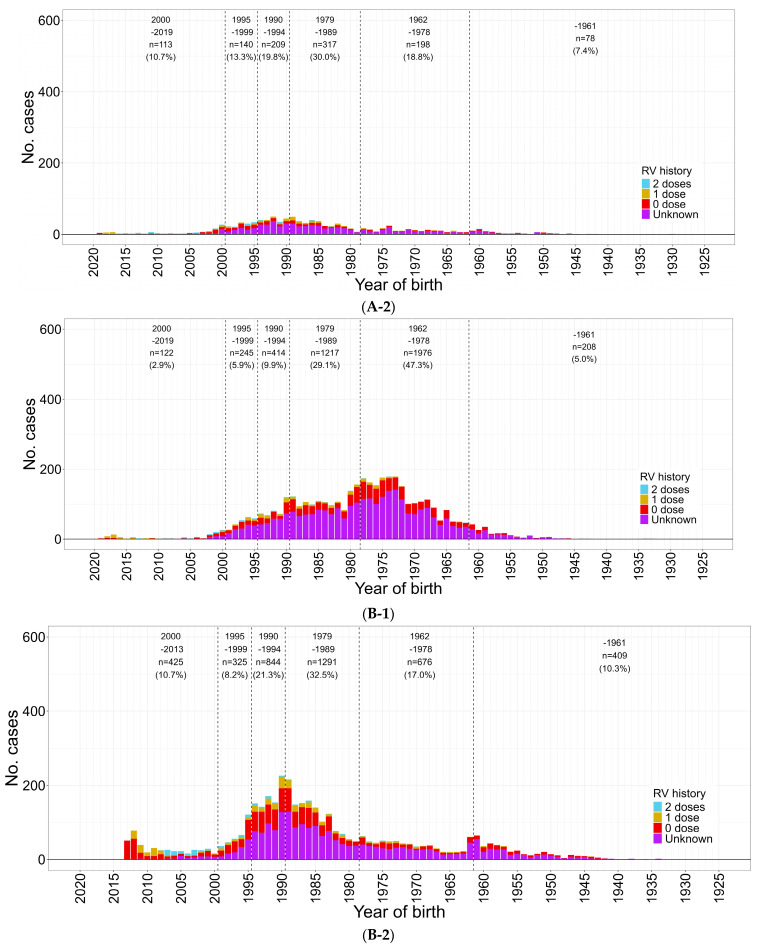
Rubella cases (confirmed) by sex, year of birth, and vaccination history during rubella outbreaks in January 2012–December 2013 and in January 2018–December 2019. (**A-1**) Male, January 2012–December 2013 (*n* = 12,765). (**A-2**) Female, January 2012–December 2013 (*n* = 3970). (**B-1**) Male, January 2018–December 2019 (*n* = 4182). (**B-2**) Female, January 2018–December 2019 (*n* = 1055). RV, rubella vaccine.

The story of progress regarding rubella elimination is different. There were two nationwide rubella outbreaks in 2012–2013 and 2018–2019 (Figure 2B and Figure 6).

During the 2012–2013 outbreak, 16,730 cases of rubella were reported. Among them, 12,763 (76.1%) were male and, among these male rubella cases, 3951 (31.0%), 5749 (45.0%), and 792 (6.2%) were from cohorts born in FY 1979–1989, FY 1962–1978, and FY 1961 and before, respectively. The male and female cohorts born after 1990 accounted for 17.7% and 40.1%, respectively (Figure 6A-1,A-2). Following this outbreak, 45 infants with CRS were identified, 12 of whom died within the first year of life [22].

During the 2018–2019 outbreak, 5237 cases of rubella were reported. Among them, 4182 (79.9%) were male and, among male cases of rubella, 1216 (29.1%), 1978 (47.3%), and 208 (5.0%) were reported from the cohorts born in FY 1979–1989, FY 1962–1978, and FY 1961 and before, respectively. The male and female cohorts born after 1990 accounted for 18.7% and 43.6% of cases, respectively (Figure 6B-1,B-2) [10,23]. Following the outbreak during 2018–2019, six infants with CRS were identified (no deaths at the time of report) [24].

### 3.6. Rubella Outbreaks in 2012–2013 and 2018–2019

**Figure 7:**
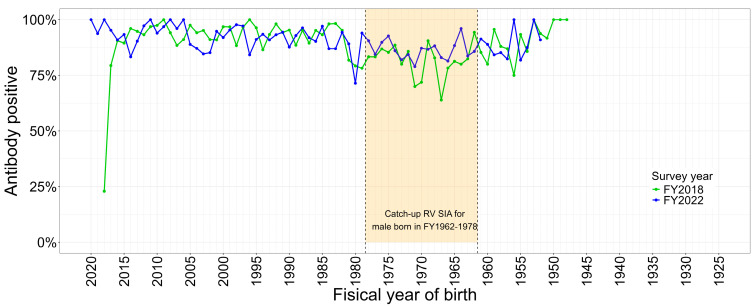
Rubella antibody positive rate (PA ≥ 1:8) among male population by year of birth in 2018 and 2022. RV, rubella-containing vaccine; SIA, supplementary immunization activity; FY, fiscal year.

The outbreaks pointed to large population rubella immunity gaps among male cohorts born in FY 1962–1989, from which 78.0% and 79.9% of male rubella cases were reported during both rubella outbreaks in 2012–2013 and 2018–2019, respectively. Male rubella cases born in FY 1962–1978 and 1979–1989 accounted for 34.3% and 23.6%, respectively, of the total rubella cases reported during the 2012–2013 outbreak and for 37.8% and 23.2%, respectively, of the total rubella cases reported during the 2018–2019 outbreak (Figure 6). NESVPD confirmed a large immunity gap among male cohorts born in 1979–1989, as well as in FY 1962–1978 (Figure 7) [7]. This assisted in identifying the group of adult males that were offered screening for rubella immunity and rubella vaccination as required on the basis of their individual serological laboratory results.

## 4. Discussion

An in-depth epidemiologic analysis of the 2007–2008 nationwide resurgence of endemic measles virus transmission supported by the case-based measles and rubella surveillance system established during the resurgence together with the national serological surveillance system revealed birth cohorts with large immunity gaps causing the nationwide measles resurgence and helped to determine susceptible age groups (i.e., children and adolescents). They were targeted through a special vaccination program for achieving measles elimination with a time-limited surge of domestic vaccine production capacity, multifaceted engagement and coordination among different levels of governments across the country, enhanced collaboration between the health and education sectors, and the strengthened outbreak response capacity of national and local government public health sectors.

Implementation of this 5-year special vaccination program, which conducted a nationwide non-selective catch-up MRV-SIAs providing school students with a one-year opportunity for two age cohorts simultaneously, successfully established high-enough population immunity among the 20 cohorts born between 1990 and 2012 to interrupt endemic transmission, enabling the country to achieve and be verified for achieving measles elimination in 2015. This nationwide program also established strong momentum for preventing measles throughout the country, which enabled Japan to achieve and sustain very high routine MRV-1 and MRV-2 coverage ongoing in all prefectures, thus preventing the reestablishment of endemic measles virus transmission following importations even during the regional measles resurgence in the Western Pacific Region in 2013–2016 and the worldwide measles resurgence in 2018–2019.

Despite this successful achievement and maintenance of measles elimination in Japan in the last 15 years, the goal announced for rubella elimination in 2014 [25] has not yet been achieved. The main difference is clearly demonstrated through the epidemiology of recent rubella outbreaks and confirmed through population serology, with the high-risk population group for rubella virus infection and transmission being adult males aged above 40 years.

The catch-up MRV-SIA launched in 2019 to achieve rubella elimination in Japan after the nationwide rubella outbreak in 2018–2019 provided an opportunity for men who were born between FYs 1962 and 1978 to receive RCV after the confirmation of insufficient immunity against rubella by low antibody titer (≦1:8) of hemagglutination inhibition (HI) (selective MRV-SIA following antibody test). The national goal for this catch-up program was to increase population immunity based on NESVPD among men born in FY 1962 to 1978 from 80% (in 2018) to 90% by the end of March 2022. However, between 2019 and November 2023, only 4.71 million men had presented for an antibody test among a total of 15.4 million men born in FY 1962–1978 (30.7%), and 1.02 million men had received RCV (6.6% of this population) [26]. With the majority of people having weekday work, a vaccination program depending on the results of antibody titer measurements would involve at least two absences from work, and thus, this population is difficult to reach.

The use of serological surveillance for measles and rubella elimination is a strategy that has been used in multiple countries. For example, the United Kingdom has used serological studies to monitor immunity levels, guide vaccination strategies, and play an important role in maintaining elimination [27]. Germany also regularly conducts serological surveys to inform public health interventions and ensure high immunity levels [28]. Japan’s approach is consistent with these international practices and demonstrates the value of continuous surveillance and adapted strategies using serological surveillance to achieve and sustain measles and rubella elimination. 

A unique strategy in Japan was the implementation of a time-phased, intensified routine vaccination program based on incidence trends, historical vaccination schedules, and serological surveillance. From the experience in Japan, to obtain effective results with the multi-year SIA method for target populations determined by the results of serological surveillance, etc., it was important that the department(s) to which the target population belonged was clear and that the active administrative approach over a long period was possible. When the expected intervention was passive and left to the voluntary efforts of individuals or groups, progress was slow. It was also considered important that the operational procedures be as flexible and simple as possible, but further research is needed.

The trends in serological surveillance for measles may suggest that a certain level of herd immunity has been formed in Japan. However, serological surveillance cannot identify individuals with insufficient or weakened immunity due to the vaccine, nor can it detect populations with low levels of immunity. From this perspective, it is important for Japan to detect and respond to individuals and groups with gaps in measles immunity, and further indicates the need for a strategy to continue maintaining a high level of herd immunity against measles through two doses of measles vaccine [29].

## 5. Limitations

This study has several limitations that need to be acknowledged. Our findings are based on the interpretation of existing data and literature, which may be subject to publication bias and the availability of studies. This means that it could involve potential underreporting and misclassification of cases. Additionally, variations in laboratory testing and reporting practices over the years may affect data consistency.

## 6. Conclusions

The target population with immunity gaps to be filled should be carefully determined before launching nationwide large-scale catch-up SIAs. Detailed epidemiological analysis of outbreaks using case-based surveillance and field investigation supported by strong laboratory networks are essential for determining this target population. National serological surveillance has been a powerful asset for planning nationwide large-scale catch-up SIAs. In countries with limited vaccine supplies but gaps in immunity, pre-screening strategies to detect people for a nationwide SIA may be an option. 

Serological surveillance can also play a pivotal role in monitoring the impact of these targeted SIAs and how successful they have been in closing the detected immunity gaps.

## Figures and Tables

**Table 1:** Number and percentage of prefectures in Japan (*n* = 47) by the coverage of measles and rubella vaccination, 2008–2022.

Vaccination	Coverage %	FY2008		FY2009		FY2010		FY2011		FY2012		FY2013		FY2014		FY2015		FY2016		FY2017		FY2018		FY2019		FY2020		FY2021		FY2022	
		*n*	%	*n*	%	*n*	%	*n*	%	*n*	%	*n*	%	*n*	%	*n*	%	*n*	%	*n*	%	*n*	%	*n*	%	*n*	%	*n*	%	*n*	%
Routine MRV-1 (the aged 1 year)	>90	45	95.7	44	93.6	47	100	46	97.9	47	100	47	100	47	100	47	100	47	100	47	100	47	100	47	100	47	100	46	97.9	46	97.9
	85–90	2	4.3	3	6.4	0	0.0	1	2.1	0	0.0	0	0.0	0	0.0	0	0.0	0	0.0	0	0.0	0	0.0	0	0.0	0	0.0	1	2.1	1	2.1
	<85	0	0.0	0	0.0	0	0.0	0	0.0	0	0.0	0	0.0	0	0.0	0	0.0	0	0.0	0	0.0	0	0.0	0	0.0	0	0.0	0	0.0	0	0.0
Routine MRV-2 (the aged 6 years)	>90	42	89.4	41	87.2	46	97.9	44	93.6	46	97.9	44	93.6	45	95.7	45	95.7	46	97.9	46	97.9	47	100	47	100	47	100	45	95.7	44	93.6
	85–90	5	10.6	6	12.8	1	2.1	3	6.4	1	2.1	3	6.4	2	4.3	2	4.3	1	2.1	1	2.1	0	0.0	0	0.0	0	0.0	2	4.3	3	6.4
	<85	0	0.0	0	0.0	0	0.0	0	0.0	0	0.0	0	0.0	0	0.0	0	0.0	0	0.0	0	0.0	0	0.0	0	0.0	0	0.0	0	0.0	0	0.0
Catch-up MRV SIA (the aged 13 years)	>90	19	40.4	19	40.4	19	40.4	25	53.2	25	53.2																				
	85–90	17	36.2	16	34.0	18	38.3	15	31.9	16	34.0																				
	<85	11	23.4	12	25.5	10	21.3	7	14.9	6	12.8																				
Catch-up MRV SIA (the aged 17 years)	>90	2	4.3	3	6.4	4	8.5	7	14.9	10	21.3																				
	85–90	9	19.1	10	21.3	15	31.9	19	40.4	22	46.8																				
	<85	36	76.6	34	72.3	28	59.6	21	44.7	15	31.9																				

MRV, measles and rubella vaccine; SIA, supplementary immunization activity; FY, fiscal year.

**Table 2:** Comparison of vaccination coverage by catch-up SIA.

A. Catch-Up SIA for Measles Elimination										
Vaccination	Year of Birth			FY 2008		FY 2009	FY 2010	FY 2011	FY 2012	Total
Catch-up MRV SIA (the aged 12–13 years)	FY 1995–1999	No. targeted		1,192,612		1,194,878	1,200,301	1,207,874	1,190,773	5,986,438
		No. vaccinated		1,015,341		1,026,892	1,047,356	1,064,727	1,057,237	5,211,553
		Coverage		85.1%		85.9%	87.3%	88.1%	88.8%	87.0%
Catch-up MRV SIA (the aged 17–18 years)	FY 1990–1994	No. targeted		1,224,084		1,213,204	1,214,161	1,201,664	1,235,125	6,088,238
		No. vaccinated		946,593		933,891	957,506	978,440	1,027,607	4,844,037
		Coverage		77.3%		77.0%	78.9%	81.4%	83.2%	79.6%
**B. Catch-Up SIA for Rubella Elimination**										
**Vaccination**	**Year of Birth**			**FY 2019**	**FY 2020**	**FY 2021**	**FY 2022**	FY 2023 *	**Other**	**Total**
Catch-up RV SIA	Male FY 1962–1978	Antibody test	No. targeted	-	-	-	-	-	-	15,374,162 ^†^
			No. tested	1,245,330	1,769,990	847,962	531,596	253,175	66,307	4,714,360
			Coverage	-	-	-	-	-	-	30.7%
		RV	No. vaccinated	270,113	359,312	200,419	121,390	55,154	13,097	1,019,485
			Coverage	-	-	-	-	-	-	6.6%

MRV, measles and rubella vaccine; RV, rubella vaccine; SIA, supplementary immunization activity; FY, fiscal year. * As of November 2023; ^†^ As of April 2019.
